# The Intervention Effects of Acupuncture on Fatigue Induced by Exhaustive Physical Exercises: A Metabolomics Investigation

**DOI:** 10.1155/2015/508302

**Published:** 2015-09-09

**Authors:** Haifeng Ma, Xia Liu, Ying Wu, Naixia Zhang

**Affiliations:** ^1^Shanghai University of Sport, Shanghai 200438, China; ^2^Department of Analytical Chemistry, Shanghai Institute of Materia Medica, Chinese Academy of Sciences, Shanghai 201203, China

## Abstract

In this study, the antifatigue effects of acupuncture had been investigated at the metabolic level on the young male athletes with exhaustive physical exercises. After a series of exhaustive physical exercises and a short-term rest, the athletes either were treated with needling acupuncture on selected acupoints (TA group) or enjoyed an extended rest (TR group). NMR-based metabolomics analysis was then applied to depict the metabolic profiles of urine samples, which were collected from the athletes at three time points including the time before exercises, the time before and after the treatment of acupuncture, or taking the extended rest. The results from multivariate statistical analysis indicated that the recoveries of disturbed metabolites in the athletes treated with acupuncture were significantly faster than in those only taking rest. After the treatment with acupuncture, the levels of distinguished metabolites, 2-hydroxybutyrate, 3-hydroxyisovalerate, lactate, pyruvate, citrate, dimethylglycine, choline, glycine, hippurate, and hypoxanthine were recovered at an accelerated speed in the TA group in comparison with the TR group. The above-mentioned results indicated that the acupuncture treatment ameliorated fatigue by backregulating the perturbed energy metabolism, choline metabolism, and attenuating the ROS-induced stress at an accelerated speed, which demonstrated that acupuncture could serve as an alternative fatigue-relieving approach.

## 1. Introduction

Fatigue is a pathological and/or physiological symptom associated with either chronic debilitating diseases [1–3] or abnormal psychological conditions [4], also being prevalent in people with exhaustive physical exercises [5, 6] and/or intensive labor works. The molecular mechanisms underlying fatigue are quite complicated, which include a systematic malfunction of metabolic pathways for energy production [7], reactive oxygen species (ROS) stress [8–10], depletion of hormones, neurotransmitters [11, 12], and other disturbance in metabolism [13]. Fatigue is usually not very harmful or life-threatening; however, under certain circumstances, severe fatigue is closely linked to the injuries happening in important organs including muscle and heart and sometimes even promotes the happening of acute attacks of serious human diseases such as heart failure and Karoshi. The relief of fatigue induced by pathological conditions or nonpathological exercises will either support the treatments of related human diseases or help to improve performances. However, only few medications show antifatigue function. Fortunately, a key component of traditional Chinese medicine, acupuncture, also shows strong antifatigue effects on mammals indicated by an increasing amount of data [14–16].

Acupuncture treatment, as one of the complementary therapeutic modalities in traditional Chinese medicine (TCM), has been frequently used to treat various human diseases including chronic pain [17], hypertension [18], Parkinson's disease [19], functional dyspepsia [20], and seasonal allergic rhinitis [21], and the practical outcomes of acupuncture have been demonstrated by quite a few of randomized controlled clinical trials [22–24]. In acupuncture-related theory, human body is covered by meridian network which is composed of 14 principal meridians and hundreds of acupoints. The stimulation of certain acupoints will help to promote function of specific organs. Although the therapeutic effects of acupuncture have been proved by a huge amount of clinical cases [25, 26] and in our previous practices [27, 28], its underlying molecular mechanisms at the metabolic level are still poorly understood. In recent decades of years, the emerging of systems biology techniques, which reveals whole genomic, proteomic, and metabolic profile changes of living systems in response to external stimuli such as drug treatments [29, 30], opens new opportunities for the working mechanism elucidations of TCM [31–33] including acupuncture [34]. Here in this paper, we used a nuclear magnetic resonance- (NMR-) based metabolomics approach to investigate the antifatigue effects of traditional acupuncture on healthy young male athletes who had done exhaustive physical exercises. In our experiments, based on the known knowledge from clinical practice [16, 35, 36] and experimental studies [14], four acupoints including* Zusanli* (ST36),* Weizhong* (BL40),* Guanyuan* (CV4), and* Shenshu* (BL23) which might have antifatigue efficacy were selected for the needling acupuncture treatment on athletes with exhaustive physical exercises. At the very beginning of the study, the athletes were randomly divided into two groups: training with acupuncture as a test group (TT group) and training without acupuncture as a control group (TC group). To induce fatigue effects, all athletes performed designed exhaustive physical exercises of a middle-distance training program. After the completion of whole exercise session, a rest for 35 minutes (min) was assigned to all runners. Then, TT group members were treated with a 30 min needling acupuncture on selected acupoints as mentioned above. In the meanwhile, during the same period, TC group athletes enjoyed an extended rest. Urine samples of athletes were collected before exercise, after whole exercise session finished and a 35 min rest taken, and after a 30 min needling acupuncture treatment done or a total of 35 min rest taken. After that, NMR measurements were done for all of the urine samples collected. Multivariate data analysis methods including principal component analysis (PCA), partial least squares-discrimination analysis (PLS-DA), and orthogonal projection of latent-structure-discrimination analysis (OPLS-DA) were applied to analyze NMR data and thus unravel possible correlations between the metabolite profile changes and the variations in biological pathways. Finally, the potential biomedical mechanism of acupuncture against fatigue was elucidated based on the multivariate statistical analysis results.

## 2. Materials and Methods

### 2.1. Materials

NaN_3_, NaH_2_PO_4_·2H_2_O, and K_2_HPO_4_·2H_2_O (all in analytical grade) were purchased from Sinopharm Chemical Reagent Co. Ltd. (Shanghai, China). D_2_O (99.9% in D) containing sodium 3-(trimethylsilyl)propionate-2,2,3,3-d_4_ (TSP) as an internal standard for chemical shift reference was provided by Sigma-Aldrich (Sigma Chemical Corp., St. Louis, MO, USA). A buffer system containing 0.2% NaN_3_ was prepared in D_2_O consisting of TSP and 1.5 M K_2_HPO_4_/NaH_2_PO_4_ (molar ratio of 4 : 1) at pH 7.4 in order to prevent pH effect on chemical shift at different concentrations.

### 2.2. Subjects

This research project was in accordance with the principle of Helsinki Declaration II and approved by the institutional ethics committee of Shanghai University of Sport. Fourteen young, moderately trained, healthy males and their caretakers provided written, informed consent for participation in the research project. The participants were randomly divided into two roughly equivalent groups: athletes with a training and following treatment of acupuncture as a test group (TT group: age, 16.2 ± 1.7 yrs.; body mass, 59.2 ± 4.4 kg; height, 173.3 ± 3.5 cm; body mass index, 19.7 ± 1.3 kg·m^−2^; fat percentage, 11.1 ± 1.6%; maximum oxygen uptake, 66.0 ± 3.0 mL·kg^−1^·min^−1^; all mean ± SEM, *n* = 7) and athletes with training and an equal-time rest as a control group (TC group: age, 16.3 ± 1.9 yrs.; body mass, 56.7 ± 4.2 kg; height, 174.9 ± 5.1 cm, body mass index 18.9 ± 0.8 kg·m^−2^; fat percentage, 10.2 ± 1.2%; maximum oxygen uptake, 66.0 ± 3.0 mL·kg^−1^·min^−1^; all mean ± SEM, *n* = 7). All the above-mentioned characteristics including age, body mass, and height have no significant differences between these two groups.

### 2.3. Exercise Section and Sample Collection

**Figure 1:**
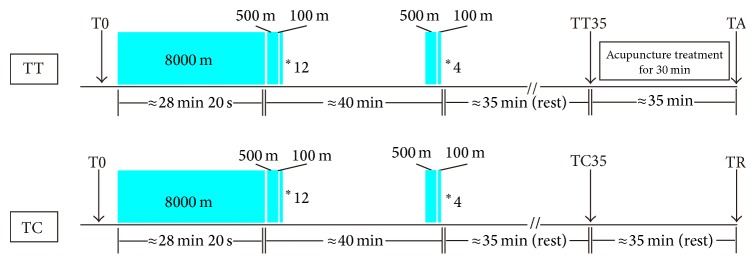
The design of the exercise session. Blocks depict 8000-meter, 500-meter, and 100-meter runs, and arrows indicate urine sampling. Min is the abbreviation of minutes.

Midstream urine samples of athletes were collected in sterile containers before the exercise as a T0 group. After finishing the warmups with dynamic stretching, each subject performed exhaustive physical exercises by following a middle-distance training program including an 8000-meter run and sixteen 500-meter running blocks at the maximum speed, each individual block spliced with one 100-meter running set at the semi-maximum speed (the runners wore regular athletic shoes in the first twelve sessions, while they wore shoes with spikes in the last four sessions). All athletes were asked to provide a rating of perceived exertion (RPE) by using the Borg Scale right before and after the excises. The heart beats of athletes were monitored during the full range of exercises. A 35-minute rest was assigned to all runners after the completion of whole exercise session, and urine samples were collected as a T35 group which was consisted of a TT35 subgroup and a TC35 subgroup. Then, the TT35 group was treated with a 30-minute needling acupuncture (classified as TA group hereafter) and the TC35 group enjoyed an extended rest (classified as TR group hereafter) for about 35 minutes. Urine samples were collected after the acupuncture treatment or the rest. Once collected, urine samples were frozen stored at −80°C until NMR analysis. The experimental design is depicted in Figure 1.

To reduce the impact of nutrient intake on metabolic profiling of the athletes during experiments, a standard dietary plan was applied during the two days before exercise testing. In this plan, carbohydrates, fats, and proteins provided 50%, 35%, and 15% of the daily calories required by individual athlete, respectively. In the morning of the experimental day, all participants ate a standardized meal. Moreover, to reduce the effect of hydration status on the urine production of athletes, all of the participants took in 0.5 L of water during the morning meal and further consumed 1 L of purified water between the preexercise and postexercise samplings.

### 2.4. Sample Preparation and ^1^H NMR Data Acquisition

Urine samples were thawed for metabolomics analysis by ^1^H NMR spectroscopy. An aliquot of 500 *μ*L from each sample was mixed with 50 *μ*L of phosphate buffer, vortexed, and then centrifuged at 12,000 g for 10 minutes at 4°C. Aliquots of the supernatant (500 *μ*L) were transferred into 5 mm diameter NMR tubes. Solvent-suppressed 1D ^1^H NOESY spectra (NoesyPr1d) were acquired using the pulse sequence [RD-90-*t*_1_-90-*t*_*m*_-90-ACQ] with a mixing time (*t*_*m*_) of 100 ms. Water resonance was suppressed by presaturation using a 25 Hz pulse during the recycle delay (RD) of 4 s and the mixing time. The 90° pulse length was adjusted to about 10.35 *μ*s. *T*_1_ was set to 4 *μ*s. A total of 4 dummy scans and 64 free induction decays (FIDs) were collected into 64 k acquisition points, covering a spectral width of 8 kHz (14 ppm) and giving an acquisition time (ACQ) of 3.00 s. All NMR experiments were carried out at 300 K on a Bruker Avance III 600 MHz spectrometer (Karlsruhe, Germany) equipped with a cryoprobe.

### 2.5. Spectral Data Processing and Multivariate Statistical Analysis

FID processing was performed using the software MestReNova Version 8.1.4 (Mestrelab Research S.L.). All 1D spectra were applied with an exponential multiplication of a 0.3 Hz line-broadening factor to improve the signal-to-noise ratio before Fourier transformation. Spectra were manually corrected for phase and baseline distortions and carefully aligned. All the 1D NMR spectra were then referenced to the methyl group of TSP at 0.00 ppm. The spectral region of *δ* 9.50–0.50 was segmented into 3000 bins with a 0.003 ppm width for each one. Every binning was labelled with its median chemical shift value. The integrals from the region of *δ* 6.30–4.70 were excluded from the analysis to eliminate the effects of distorted baseline from imperfect water saturation and urea signal. The integrals of remaining binning will be normalized to 1 and used as a dataset in the following multivariate analysis. In another dataset, because lactate was the most significant variance in the model of T0 versus T35 and strongly affected the clustering of groups in the multivariate significant scores plots, the integrals of *δ* 1.220–1.241, *δ* 1.321–1.344, 1.420–1.453, and *δ* 4.100–4.160 mainly with the signals of lactate and its' satellite peaks were removed from the analysis. Subsequently, the normalized integral values were mean centered for PCA, PLS-DA, and OPLS-DA by SIMCA-P+ software package (Version 12.0, Umetrics AB, Umeå, Sweden). The unbiased tool of PCA was used to detect the intrinsic clustering. Then the supervised method of PLS-DA was employed to study the contribution of the variables in group separation. Additionally, the OPLS-DA method applied an orthogonal filter to remove the variations unrelated to the group separation and thus identify the most significant variations which contributed to the differentiation between groups.

To assess the cross-validated prediction power of the model and avoid overfitting the PLS-DA model, the 7-round (T35 versus T0 model) or 6-round (TT35 versus TC35 and TA versus TR models) cross-validation and permutation tests (999 cycles, by the usual leave-one-out device) were carried out to measure the robustness of models [37]. Moreover, to determine the value of sensitivity and specificity, the receiver operating characteristic (ROC) maps were established in the R-studio software (Version 0.98) to assess the prediction performance of the model. The area under the ROC curve (AUC) was calculated at the same time to evaluate the model fitness, where a clustering with the value of AUC greater than the threshold indicated better chance agreement.

The correlation coefficients of the variables relative to the first predictive component in the OPLS-DA model were extracted from S-plot. Cut-off values with the significant level of *p* = 0.05 were used to identify variables that were responsible for the discrimination of the groups [38]. Variable importance in the projection (VIP) values was also employed to identify the differentiating biomolecules with the VIP value higher than 1, which contributed significantly to the model clustering. Therefore, we chose those variables meeting twofold criteria (|*r*| ≥ the cut-off value of *p* = 0.05, and VIP ≥ 1) as the most significant and reliable variables which were responsible for the separation of the clusters.

### 2.6. Quantitative Comparison of Metabolites in ^1^H NMR Spectra of Urine Samples

The average changes of metabolites between TA versus TR, T35 versus T0, T35 versus T0 (the signals of lactate excluded), TA versus T0, and TR versus T0 groups were calculated [39]. The box charts were used to depict the variation in the integrals of the most significant metabolites in T0, T35, TA, and TR groups. Significant differences in the mean values were evaluated by one-way analysis of variance (ANOVA), followed by Bonferroni's or Turkey's post hoc analysis when appropriate. Statistical significance was considered at *p* ≤ 0.05. All the statistical analysis was performed using SPSS software (Version 17.0, SPSS, Chicago, IL).

## 3. Results

### 3.1. Ratings of Perceived Exertion

All athletes performed exhaustive physical exercises, and during the full range of exercises their average heart rate reached a value of 169 ± 12.6 beats per minute. After the exercises, all of the athletes were exhausted and their Borg's ratings of perceived exertion [40] sharply changed from 7-8 to 19-20.

### 3.2. ^1^H NMR Spectra

Representatives of four one-dimensional ^1^H NMR spectra of urine samples obtained from T0, T35, TA, and TR groups are depicted in Figure S1 as shown in the Supplementary Material (see Supplementary Materials available online at http://dx.doi.org/10.1155/2015/508302). The metabolite resonances were annotated based on chemical shifts, proton-proton coupling constants, 2D NMR spectra, and published literatures [41–43]. The assignments (both chemical shift and multiplicity) of metabolites are summarized in Table S1 as shown in the Supplementary Material. NMR spectra of urine samples were dominated by numerous signals from amino acids (leucine, isoleucine, valine, alanine, glutamine, glycine, N-phenylacetylglycine, and tyrosine), carboxylic acids (2-hydroxyisovalerate, 2-hydroxybutyrate, isocaproate, 2-aminobutyrate, isobutyrate, 3-aminoisobutyrate, 3-hydroxyisovalerate, pyruvate, succinate, citrate, fumarate, formate, and lactate), methylamine metabolites (2-dimethylamine, trimethylamine, trimethylamine N-oxide (TMAO), methylguanidine, and dimethylglycine), the nitrogen containing heterocycle molecules (N-methylnicotinamide (NMN), trigonelline (TRG), hypoxanthine (Hyx), and creatinine), N-acetyl-glycoprotein, taurine, hippurate, and so on. The characterization of metabolic profiles based on such complicated spectra could be greatly facilitated by multivariate statistical analysis, such as PCA, PLS-DA, and OPLS-DA. Thus, the datasets of binned spectra were imported into the SIMCA-P+12.0 software package for the multivariate analysis.

### 3.3. Reliability Assessment of Subject Grouping Applied in This Study

In order to determine whether any bias for the selection of candidates was incorporated in the program, multivariate statistical analysis was carried out for the TT35 versus TC35 groups (see Supplementary Material Figure S2). The PCA (see Supplementary Material Figure S2A), PLS-DA (see Supplementary Material Figure S2B), and OPLS-DA (see Supplementary Material Figure S2C) scores plots of TT35 versus TC35 showed no clues for the discrimination between these two groups. Moreover, the permutation tests (see Supplementary Material Figure S2D) also revealed that the PLS-DA model (see Supplementary Material Figure S2B) of TT35 versus TC35 was overfitting, which was indicated by the observations that the *Q*2 values to the left were higher than the original point to the right. The results mentioned above supported that there was no metabolic bias between TT35 and TC35 groups, which made the following assessment of antifatigue effects of acupuncture on the young male athletes with exhaustive physical exercises reliable.

### 3.4. The Change Trend of Metabolic Profiles in the T0, TA, and TR Groups

**Figure 2:**
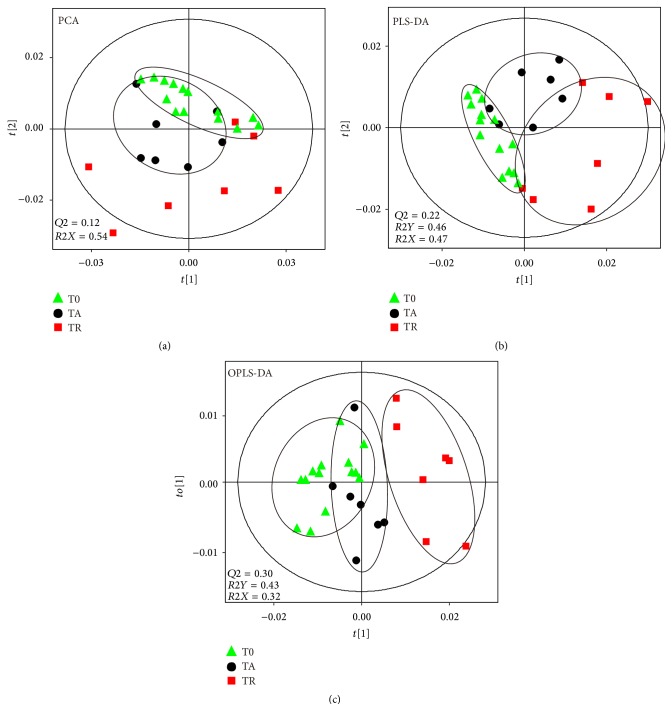
Scores plots of PCA (a), PLS-DA (b), and OPLS-DA (c) derived from ^1^H NMR data of TA, TR, and T0 groups. Variables of scores plots are centered and scaled.

**Figure 3:**
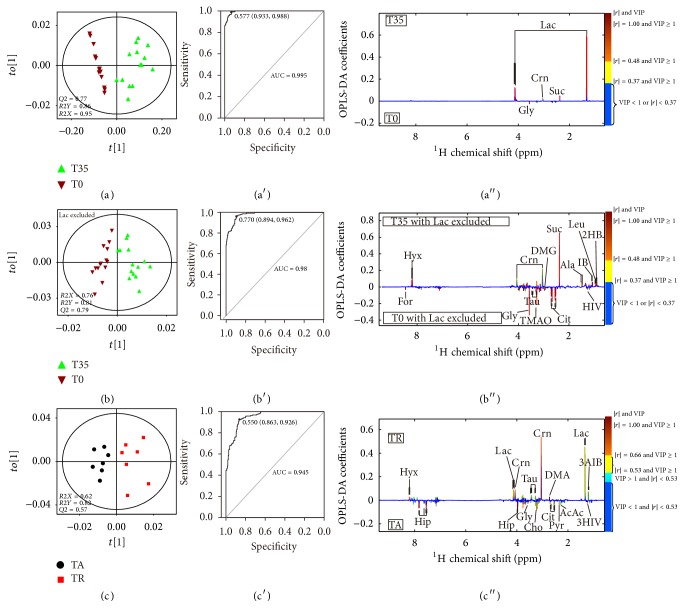
OPLS-DA scores plots of ^1^H NMR data, ROC maps, and OPLS-DA coefficient plots of ^1^H NMR data derived from PLS-DA models of T35 versus T0 groups (a, a′, and a′′), T35 versus T0 groups (b, b′, and b′′) with lactate excluded, and TA versus TR group (c, c′, and c′′). The abbreviations of metabolites are denoted in Table S1 as shown in the Supplementary Material.

Unbiased clustering and unsupervised separation among T0, TA, and TR groups were illustrated by the PCA scatter plot (Figure 2(a)), showing some overlapping among metabolic profiles. However, the degree of dispersion of data points among groups in the PCA scores plot was clear and unambiguous, that is, TR > TA > T0. In order to increase the separation among groups, supervised methods of PLS-DA (Figure 2(b)) and OPLS-DA (Figure 3(b)) models were established, showing clear group discriminations and facilitating easy interpretation. In particular, in the OPLS-DA scores plots, the metabolic profiles of TR and T0 were clearly separated in the first predictive component (*t*[1]), indicating a severe metabolic perturbation induced by ischemia-reperfusion injury. In addition, the metabolic pattern of TA moved closer towards the T0 group than the TR group indicating that the efficacy of acupuncture attenuated urine metabolic disturbance at an accelerating rate compared with the rest.

### 3.5. Metabolic Evaluation of Fatigue Effects Induced by Exhaustive Physical Exercises

To investigate the metabolic disturbance induced by exhaustive physical exercises, the methods of PCA (see Supplementary Material Figure S3A), PLS-DA (see Supplementary Material Figure S3A′), and OPLS-DA (Figure 3(a)) between T35 and T0 groups were carried out. The result showed that the postexercise samples (T35) were metabolically differentiated from the preexercise samples (T0) basically by the first component. Moreover, the permutation tests (see Supplementary Material Figure S3A′′) strongly indicated that the PLS-DA model (Figure 3(b′)) of T35 versus T0 was valid, which was shown by the observations that the *Q*2 regression line had a negative intercept and *Q*2 values to the right were higher than the original point to the left. The robustness of the separation between T35 and T0 groups was also verified by ROC curve (Figure 3(b′)). The predictive results of ROC curve showed 93.3% specificity, 98.8% sensitivity, and 0.985 area under the curve, indicating a good predictive power of the original model. Postexercise samples showed a higher extent of dispersion, indicating the different responses of the individuals to strenuous physical exercises. However, according to the coefficient numbers and the VIP values obtained, only a few of altered metabolites were identified to account for the group separation. Compared with the T0 samples, the prominent changes in the T35 samples were the increased levels of lactate and succinate, and the decreased level of glycine (Figure 3(a′′)). In order to get a better view of the contributions from other metabolites, the extremely strong lactate signals and its ^13^C satellite peaks signals were removed from the statistical analysis. The resulting dataset still provided a clear-cut separation of preexercise and postexercise samples in PCA (see Supplementary Material Figure S3B), PLS-DA (see Supplementary Material Figure S3B′), and OPLS-DA models (Figure 3(b)). The R2X values (0.76), R2Y values (0.81), *Q*2 values (0.79), the permutation test result (see Supplementary Material Figure S3B′′), and the ROC map (Figure 3(b′)) indicated that the robustness and the predictive power of discrimination analysis model were satisfying. Several discriminating metabolite markers were revealed in the coefficient plot of T35 versus T0 with lactate excluded. Compared with the T0 samples, the levels of 2-hydroxybutyrate, leucine, isobutyrate, alanine, succinate, and hypoxanthine were upregulated in the T35 samples. Meanwhile, the levels of certain metabolites including 3-hydroxyisovalerate, pyruvate, glutamine, citrate, dimethylglycine, trimethylamine N-oxide, taurine, glycine, hippurate, and formate declined in the T35 samples (Figure 3(b′′) and Table 1).

### 3.6. Antifatigue Effects of Acupuncture

**Figure 4:**
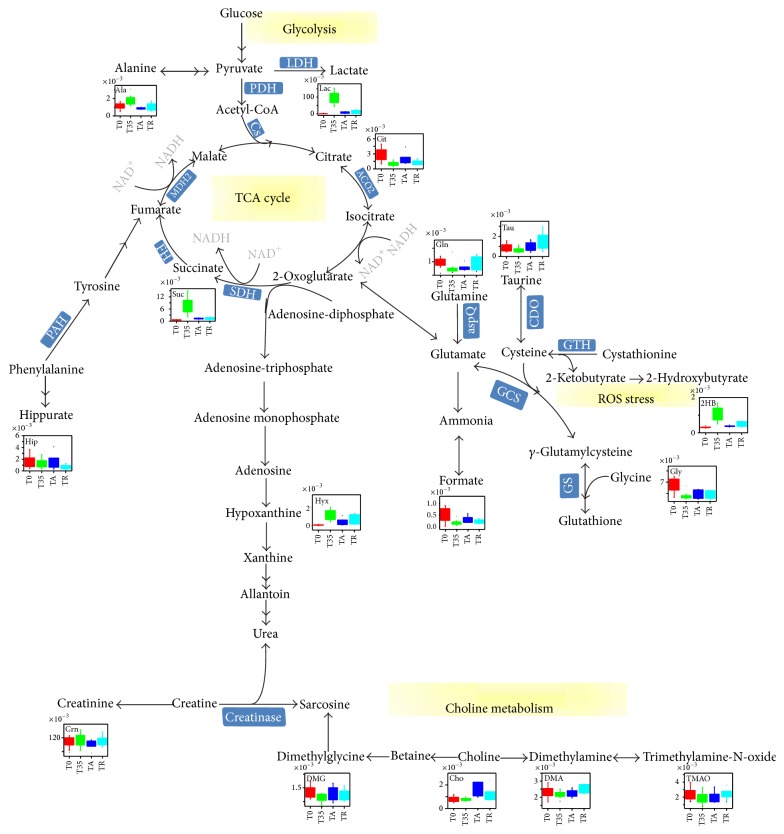
Potential metabolic pathways disturbed in the T35 group and altered in the TA and TR groups. The level changes of significant metabolites were depicted by their respective box chart.

After the treatment of acupuncture, a clear separation between the TA group and the TR group was observed in the PCA (see Supplementary Material Figure S3C), PLS-DA (see Supplementary Material Figure S3C′), and OPLS-DA (Figure 3(c)) score plots. Besides, in comparison with the TR samples, the cluster of TA group moved backward and became close to that of T0 group (Figure 2), indicating that the recoveries of the athletes in TA group were significantly faster than those without acupuncture treatment. Compared with those metabolites in the TR samples, the excretion of 3-aminoisobutyrate, lactate, dimethylamine, creatinine, taurine, and hypoxanthine in the TA samples was decreased, and these variations were accompanied by the increased levels of 2-hydroxyisovalerate, pyruvate, citrate, glycine, and hippurate. Referring to the Kyoto Encyclopedia of Genes and Genomes (KEGG) Database [44] and the Human Metabonome Database (HMDB) [45], these altered metabolites were finally connected to different pathways and key enzymes (Figure 4). Compared to the T0 group (see Supplementary Material Figure S4), according to the average change percentages of metabolites in comparisons of TA versus T0 and T35 versus T0 (Table 1), the level changes of alanine (−26.4% for TA versus T0 and +56.0% for T35 versus T0), succinate (+124.2% for TA versus T0 and +1434.8% for T35 versus T0), taurine (+3.4% for TA versus T0 and −28.5% for T35 versus T0), lactate (+456.5% for TA versus T0 and +7770.5% for T35 versus T0), citrate (−33.6% for TA versus T0 and −64.0% for T35 versus T0), pyruvate (+32.7% for TA versus T0 and −20.0% for T35 versus T0), dimethylglycine (−2.2% for TA versus T0 and −6.6% for T35 versus T0), 2-hydroxybutyrate (+20.4% for TA versus T0 and +242.2% for T35 versus T0), 3-hydroxyisovalerate (+2.9% for TA versus T0 and −56.5% for T35 versus T0), and hypoxanthine (+648.9% for TA versus T0 and +2000.0% for T35 versus T0) indicated that the metabolic profiles of athletes with the treatment of acupuncture partially recovered to their initial states (before taking exercises). The above-mentioned results were supported by the data that in comparison with the TC group the clustering of TA group was closer to the T0 group in the OPLS-DA model (Figure 2(c)).

## 4. Discussion

Fatigue is a symptom linked to either pathological conditions or exhaustive exercises/labor works. It is usually not life-threatening. However, under certain circumstances, fatigue can be a sign of a serious mental and/or physical condition. Nonmedical methods including more sleep, better nutrition, and less stressful environment can be applied to relieve fatigue symptoms [46, 47]. When these methods do not work well or are not applicable, alternative therapies including conventional medicines or TCMs can be used. It is worth noting that different from the situations of conventional medicines, the therapeutic effects of TCMs including those with antifatigue effects are much less proved by experimental evidences. Here in our paper, to elucidate the underlying systematic molecular mechanisms of the antifatigue effects presented by acupuncture (a key component of TCM), an NMR-based metabolomics approach was applied to investigate the intervention effects of acupuncture on the young male athletes with exhaustive physical exercises.

Since fatigue is caused by lack of energy and motivation, its underlying molecular mechanisms usually include a systematic dysfunction of metabolic pathways for energy production and ROS (reactive oxygen species) stress caused by abnormal generation of reactive oxygen species [9, 10]. Consistent with the above-mentioned facts, in the urine samples of athletes who had finished the intensive exercises (T35), the levels of key metabolites in the Krebs cycle and glycolysis such as lactate, succinate, pyruvate, and citrate were significantly perturbed (Table 1 and Figures 3(a) and 3(b)). The exhaustive exercises led to a significant increase of the energy consumption of human body and a coupled insufficient oxygen supplying. The ATP molecules required by the exercises were then produced mainly by glycolysis instead of aerobic oxidation of glucose. A huge amount of lactate, which was a product of glycolysis, was generated (Table 1). Meanwhile, the levels of a couple of key intermediates of the Krebs cycle (core machinery of glucose aerobic oxidation) including pyruvate and citrate were downregulated. Moreover, the burst of glycolysis triggered ROS stress which in turn caused the observed perturbations of ROS-related metabolites including 2-hydroxybutyrate, hypoxanthine, and succinate in the urine samples of athletes with exhaustive exercises (T35 versus T0, Table 1). 2-Hydroxybutyrate was produced as a by-product of the pathway from methionine to key antioxidant reagent glutathione [48]. As ROS stress increased in athletes who had done strenuous exercises, glutathione synthesis was enhanced and so was the production of 2-hydroxybutyrate. Moreover, since reactive oxygen species H_2_O_2_ would be produced via the conversion from hypoxanthine to xanthine, increased level of uric hypoxanthine was also a sign of ROS stress in the bodies of athletes [49, 50]. Different from either the situation of 2-hydroxybutyrate or that of hypoxanthine, observed accumulation of succinate was mainly caused by the inhibition of the activity of succinate dehydrogenase (SDH), which had been reported to function as a ROS sensor for ROS stress [51]. Our above-mentioned dysfunctions of metabolic pathways for energy production and ROS stress have also been extensively observed in exercise metabolomics studies reported previously [52–56]. In fact, although metabolic profile changes might vary with different exercise training procedure applied, the disturbed energy metabolism (glycolysis, TCA cycle intermediates) and oxidative stress were the overwhelming phenotype characteristics of observed metabolic perturbations induced by physical exercises [52–57].

All of the above-mentioned changes of the energy production-related metabolites and the ROS stress-related metabolites indicated that the athletes who had finished the intensive exercises and taken a rest lasting for 35 minutes were definitely in fatigue state. In the meanwhile, the metabolomics analysis data also proved that both the extended rest (TR group) and the acupuncture treatment (TA group) ameliorated the fatigue effects observed in athletes (Figure 3, Table 1). However, it was worth noting that the recoveries of the athletes in TA group were significantly faster than those without acupuncture treatment. In comparison with the TR samples, the cluster of TA group moved backward further and became closer to that of T0 group (Figure 2), and the extent of recovery for quite a few of the metabolites perturbed by exercises including lactate, succinate, citrate, hypoxanthine, hippurate, and formate was significantly larger in TA group (Table 1, Figure 3). More interestingly, needling acupuncture treatment did not only present intervention effects by backregulating most of the perturbed metabolites toward normal but also presented them by overregulating metabolite including choline to reach a level higher than normal (Table 1, Figure 3). Both choline and dimethylglycine are involved in choline metabolism which is partially tuned by the functional display of intestinal bacteria. The upregulations of these two metabolites in athletes with acupuncture treatment indicated that needling acupuncture modulated the metabolic patterns of gut bacteria.

In summary, based on ^1^H NMR spectra of urine samples, we could identify the metabolic profiles of the exhaustively exercised athletes treated with or without needling acupuncture. In comparison with the normal rest, the acupuncture treatment ameliorated fatigue effects by backregulating the perturbed energy metabolism and choline metabolism and relieving the induced ROS stress at an accelerated speed. Our work revealed the underlying biochemical mechanism of the antifatigue effects presented by acupuncture on selected acupoints and provided the supporting experimental data at the metabolic level for the possible application of acupuncture in sports.

## Supplementary Material

For the sake of clarity of the manuscript and providing the supportive material for the results and discussion, we included five figures and two tables in the supplementary material file. Figure S1 displayed the assignments of metabolites in 1H-NMR spectra recorded for the T0, T35, TA, and TR group samples, in which, the abbreviations of metabolic compounds and the detail 1H chemical shifts (ppm) of each compound were referred to Table S1. Meanwhile, the mean integrals of metabolites for each group were listed in Table S2. The prominent changes among the spectra were the signals of hypoxanthine and lactate (Figure S1). However, since other subtle changes were not distinguished visibly, the metabolic profile analysis was applied to pair-wised groups, including TT35 versus TC 35 (Figure S2A, B, and C), T35 versus T0 (Figure S3A, A'), T35 versus T0 with the signals of lactate excluded (Figure S3B, B'), TA versus TR (Figure S3C, C'), TA versus T0 (Figure S4A, B, C), TR versus T0 (Figure S4E, F, G), TA versus T35 (Figure S4A', B', C'), and TR versus T35 (Figure S4E', F', G'). All the PLS-DA models were validated by permutation tests with the 6-round cross validation to test the robustness. Except the model of TT35 versus TC35 (Figure S2D), other models (Figure S3A", B", C", Figure S4D, H, D', H') were demonstrated to be valid. In the end, the metabolites contributed to the clustering differentiation of TA versus T0, and TR versus T0 were displayed in two coefficient-loading plots (Figure S5).

## Figures and Tables

**Table 1:** The change trends of discriminitive metabolites between groups.

Metabolites	*δ* ^1^H (ppm)	% average changes in TA relatively to TR (|*r*|, VIP, *p*) (*r* = 0.532)	% average changes in T35 relatively to T0 (|*r*|, VIP, *p*) excluding Lac (*r* = 0.404)	% average changes in T35 relatively to T0 (|*r*|, VIP, *p*) (*r* = 0.404)	% average changes in TA relatively to T0 (|*r*|, VIP, *p*) (*r* = 0.433)	% average changes in TR relatively to T0 (|*r*|, VIP, *p*) (*r* = 0.433)
2HB	0.90	−19.3 (0.46, 0.24, 0.10)	+242.2 (0.93, 2.81, 0.00)	/	+20.4 (0.67, 0.20, 0.00)	+49.2 (0.81, 0.42, 0.02)
Leu	0.95	/	+31.3 (0.91, 1.36, 0.00)	/	/	/
IB	1.08	/	+27.4 (0.70, 1.20, 0.02)	/	/	/
3AIB	1.20	−43.1 (0.20, 2.08, 0.16)	/	/	−17.6 (0.18, 1.12, 0.73)	+44.9 (0.22, 1.44, 0.46)
3HIV	1.24	+5.6 (0.66, 1.80, 0.28)	−56.5 (0.56, 1.47, 0.00)	/	+2.9 (0.28, 1.47, 0.80)	−2.6 (0.23, 0.97, 0.78)
Lac	1.34	−46.4 (0.56, 16.2, 0.03)	/	+7770.5 (0.97, 27.9, 0.00)	+456.5 (0.90, 19.2, 0.00)	+932.2 (0.72, 25.3, 0.00)
Ala	1.48	/	+56.0 (0.50, 1.84, 0.02)	/	−26.4 (0.60, 1.30, 0.19)	−14.7 (0.24, 1.16, 0.23)
Pyr	2.35	+73.7 (0.43, 2.36, 0.07)	−20.0 (0.26, 1.07, 0.17)	/	+32.7 (0.27, 1.35, 0.20)	−23.6 (0.21, 0.53, 0.15)
Suc	2.38	/	+1434.8 (0.91, 30.0, 0.00)	/	+124.2 (0.90, 2.03, 0.00)	+121.4 (0.88, 1.38, 0.01)
Gln	2.45	/	−25.7 (0.72, 1.69, 0.00)	/	−23.4 (0.61, 1.21, 0.01)	−14.5 (0.44, 0.79, 0.12)
Cit	2.55	+52.5 (0.48, 1.88, 0.07)	−64.0 (0.68, 8.26, 0.00)	/	−33.6 (0.54, 4.97, 0.04)	−56.5 (0.56, 6.07, 0.00)
DMA	2.72	−14.7 (0.45, 1.06, 0.02)	−6.6 (0.36, 0.99, 0.18)	/	−2.2 (0.29, 0.48, 0.35)	+14.7 (0.18, 0.70, 0.10)
DMG	2.93	+11.2 (0.09, 0.32, 0.26)	−29.8 (0.48, 1.83, 0.000)		−10.4 (0.42, 0.67, 0.21)	−19.5 (0.34, 0.84, 0.04)
Crn	3.04	−6.2 (0.67, 25.9, 0.04)	/	+3.9 (0.07, 8.26, 0.20)	−3.5 (0.06, 4.89, 0.23)	+2.9 (0.03, 10.5, 0.292)
Cho	3.21	+83.9 (0.17, 2.68, 0.12)	−3.6 (0.06, 0.26, 0.34)	/	+167.8 (0.44, 3.59, 0.06)	+45.6 (0.46, 1.30, 0.07)
TMAO	3.27	−18.4 (0.19, 1.25, 0.10)	−31.5 (0.51, 3.63, 0.03)	/	−24.8 (0.25, 7.56, 0.09)	−7.8 (0.20, 4.02, 0.33)
Tau	3.43	−37.3 (0.24, 1.52, 0.08)	−28.5 (0.47, 1.23, 0.02)	/	+3.4 (0.23, 0.61, 0.44)	+64.9 (0.33, 2.22, 0.07)
Gly	3.57	+14.9 (0.33, 1.12, 0.11)	−60.0 (0.82, 17.2, 0.000)	−60.0 (0.82, 17.2, 0.000)	−53.0 (0.74, 11.98, 0.00)	−59.1 (0.43, 11.87, 0.00)
Hip	7.83	+176.2 (0.75, 3.17, 0.01)	−21.7 (0.17, 0.99, 0.26)	/	−18.8 (0.08, 0.31, 0.28)	−70.6 (0.33, 3.66, 0.01)
Hyx	8.19	−46.2 (0.55, 1.02, 0.01)	+2000.0 (0.87, 4.49, 0.00)	/	+648.9 (0.79, 1.23, 0.02)	+1292.6 (0.78, 2.54, 0.00)
For	8.46	/	−69.6 (0.57, 1.64, 0.00)	/	−48.0 (0.51, 0.95, 0.01)	−57.7 (0.45, 0.92, 0.00)

“+” means an increase.

“−” means a decrease.

“/” means no significant change (|*r*| < the cut-off value, VIP < 1 and/or *p* ≥ 0.05).

^a^The absolute values of correlation number extracted from the correlation plots of OPLS-DA models. The cut-off values are 0.532 in the coefficient plot of TA versus TR, 0.404 in the coefficient plot of T35 versus T0, and 0.433 in the coefficient plot of TA versus T0 and TR versus T0.

^b^The *p* values were obtained from one-way ANOVA.
