# Supplementary material for: The Intervention Effects of Acupuncture on Fatigue Induced by Exhaustive Physical Exercises: A Metabolomics Investigation
Source: Evid Based Complement Alternat Med. 2015 Sep 9;2015:508302. doi: 10.1155/2015/508302 (PMC4579316; doi:10.1155/2015/508302)
Supplement: Supplementary file 1 — For the sake of clarity of the manuscript and providing the supportive material for the results and discussion, we included five figures and two tables in the supplementary material file. Figure S1 displayed the assignments of metabolites in 1H-NMR spectra recorded for the T0, T35, TA, and TR group samples, in which, the abbreviations of metabolic compounds and the detail 1H chemical shifts (ppm) of each compound were referred to Table S1. Meanwhile, the mean integrals of metabolites for each group were listed in Table S2. The prominent changes among the spectra were the signals of hypoxanthine and lactate (Figure S1). However, since other subtle changes were not distinguished visibly, the metabolic profile analysis was applied to pair-wised groups, including TT35 versus TC 35 (Figure S2A, B, and C), T35 versus T0 (Figure S3A, A'), T35 versus T0 with the signals of lactate excluded (Figure S3B, B'), TA versus TR (Figure S3C, C'), TA versus T0 (Figure S4A, B, C), TR versus T0 (Figure S4E, F, G), TA versus T35 (Figure S4A', B', C'), and TR versus T35 (Figure S4E', F', G'). All the PLS-DA models were validated by permutation tests with the 6-round cross validation to test the robustness. Except the model of TT35 versus TC35 (Figure S2D), other models (Figure S3A", B", C", Figure S4D, H, D', H') were demonstrated to be valid. In the end, the metabolites contributed to the clustering differentiation of TA versus T0, and TR versus T0 were displayed in two coefficient-loading plots (Figure S5). [file 508302.f1.pdf]

Supplementary figures and tables

Figure S1. Four typical 600 MHz ^{1}H NMR noesyPr1d spectra (δ 0.6-4.7, 6.3-9.4) of urine samples from athletes of T0 (A), T35 (B), TA(C), and TC (D) groups.

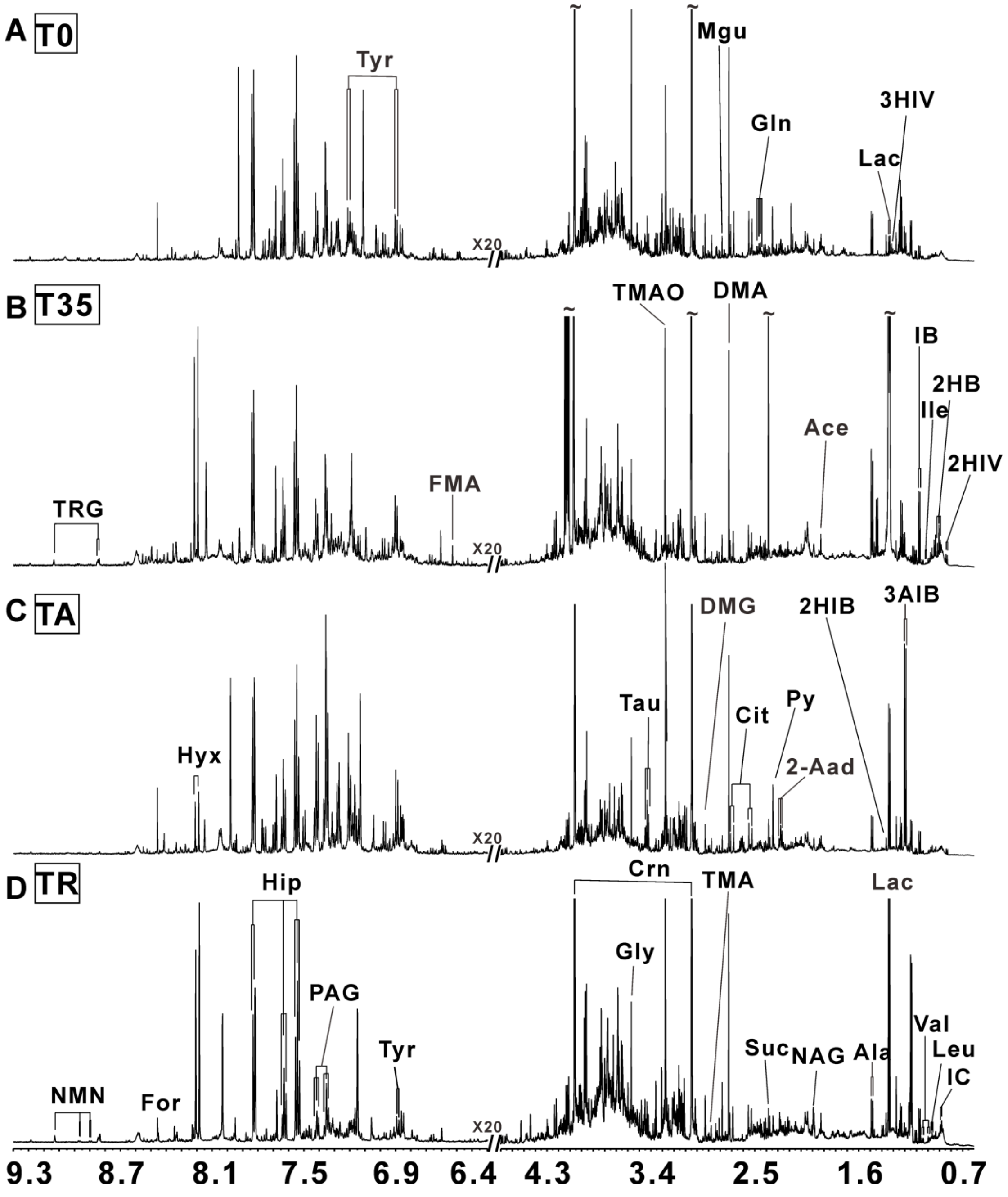

Figure S2. PCA (A), PLS-DA (B), OPLS-DA (C) scores plots of ^{1}H NMR data of TT35 and TC35 groups, and permutation tests (D) derived from PLS-DA models with the 6-round cross validation.

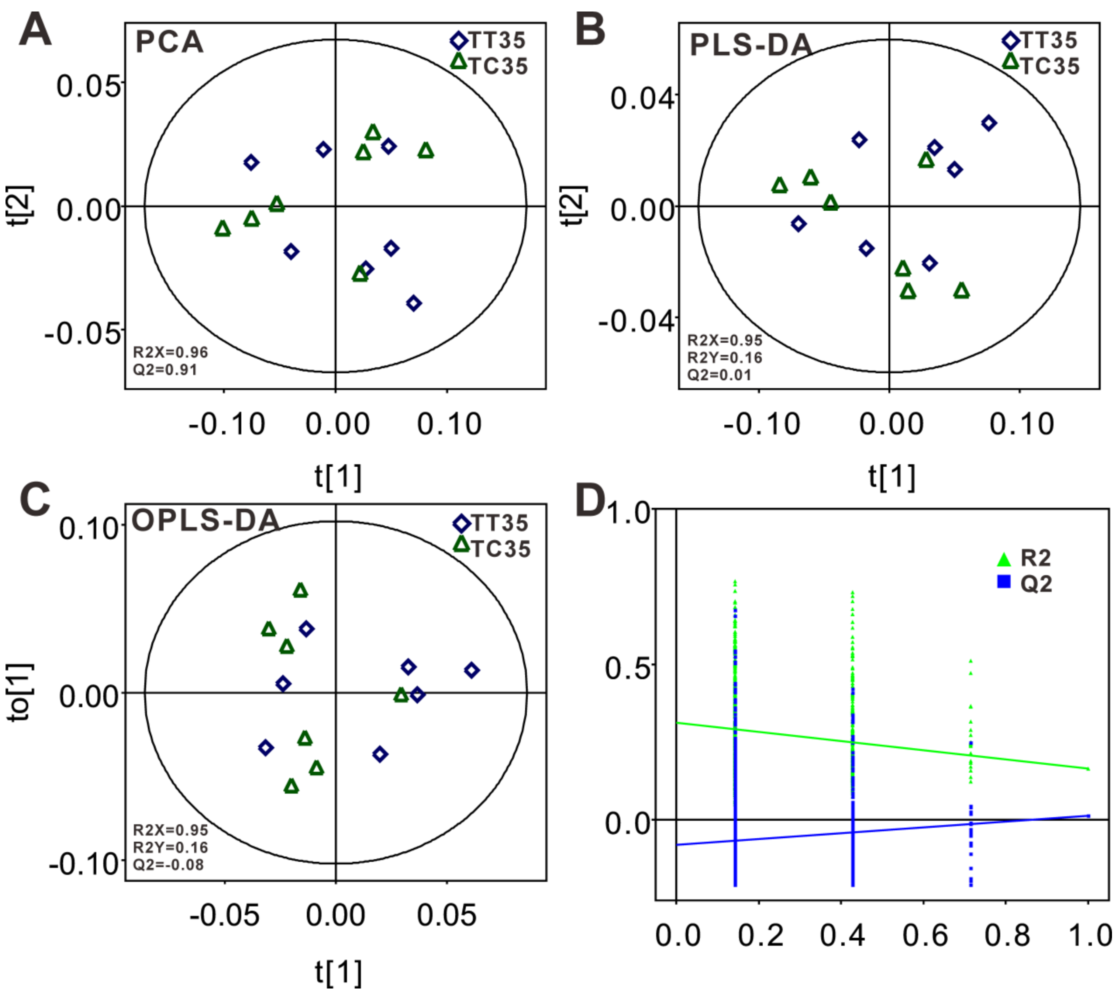

Figure S3. Scores plots of PCA and PLS-DA derived from ^{1}H NMR data, and permutation tests derived from PLS-DA models with the 6-round cross validation of T35 vs. T0 groups (A, A', A''), T35 vs. T0 groups with the signals of lactate excluded (B, B', B''), and TA vs. TR groups (C, C', C''). Variables of scores plots are centered and scaled.

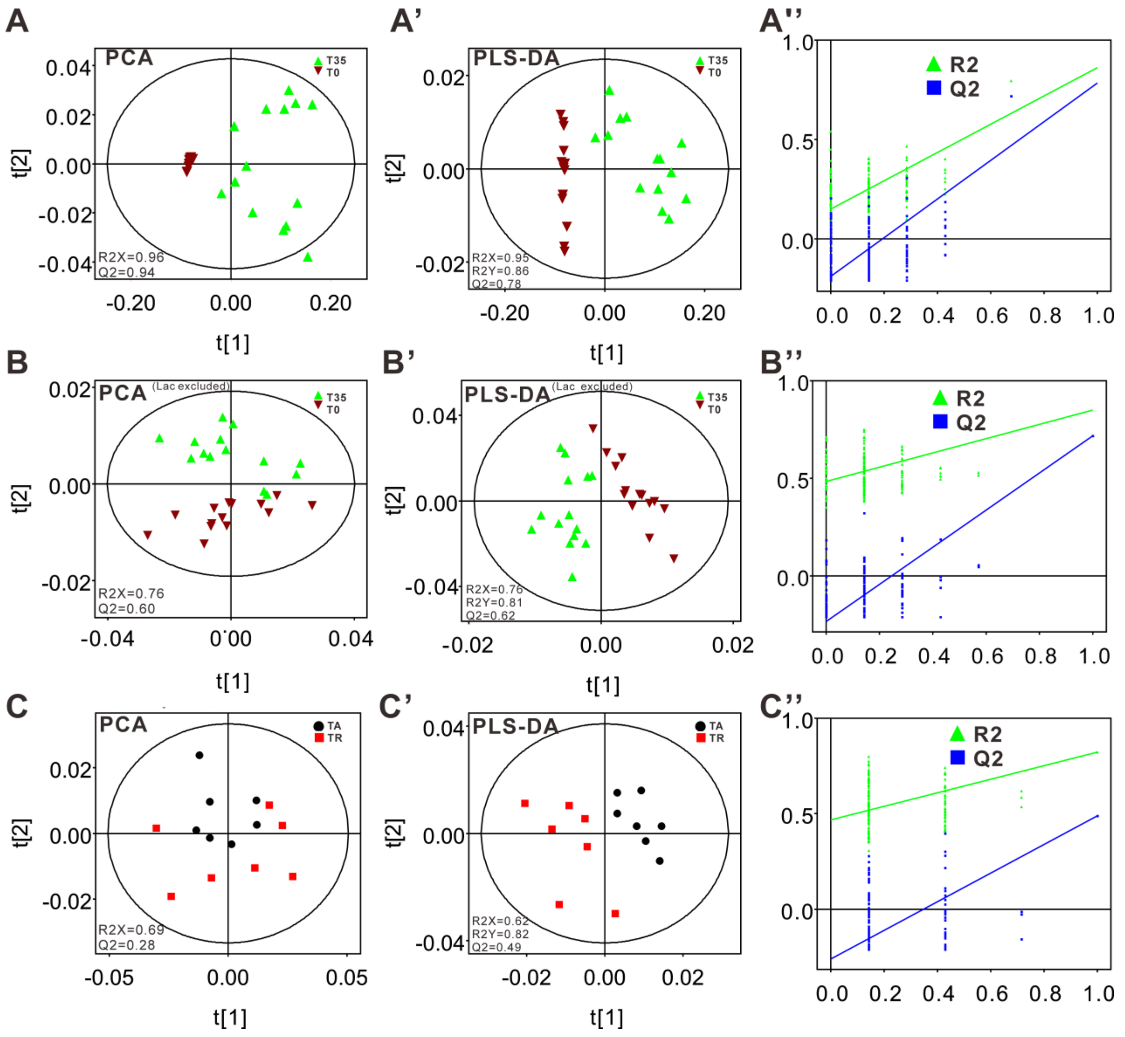

Figure S4. Scores plots of PCA, PLS-DA, OPLS-DA derived from ^{1}H NMR data of TA vs. T0 groups (A, B, C), TR vs. T0 groups (E, F, G), TA vs. T35 groups (A’, B’, C’), TR vs. T35 (E’, F’, G’) groups, and permutation tests of TA vs. T0 groups (D), TR vs. T0 groups (H), TA vs. T35 groups (D’), TR vs. T35 groups (H’) derived from PLS-DA models with the 6-round cross validation. Variables of scores plots are centered and scaled.

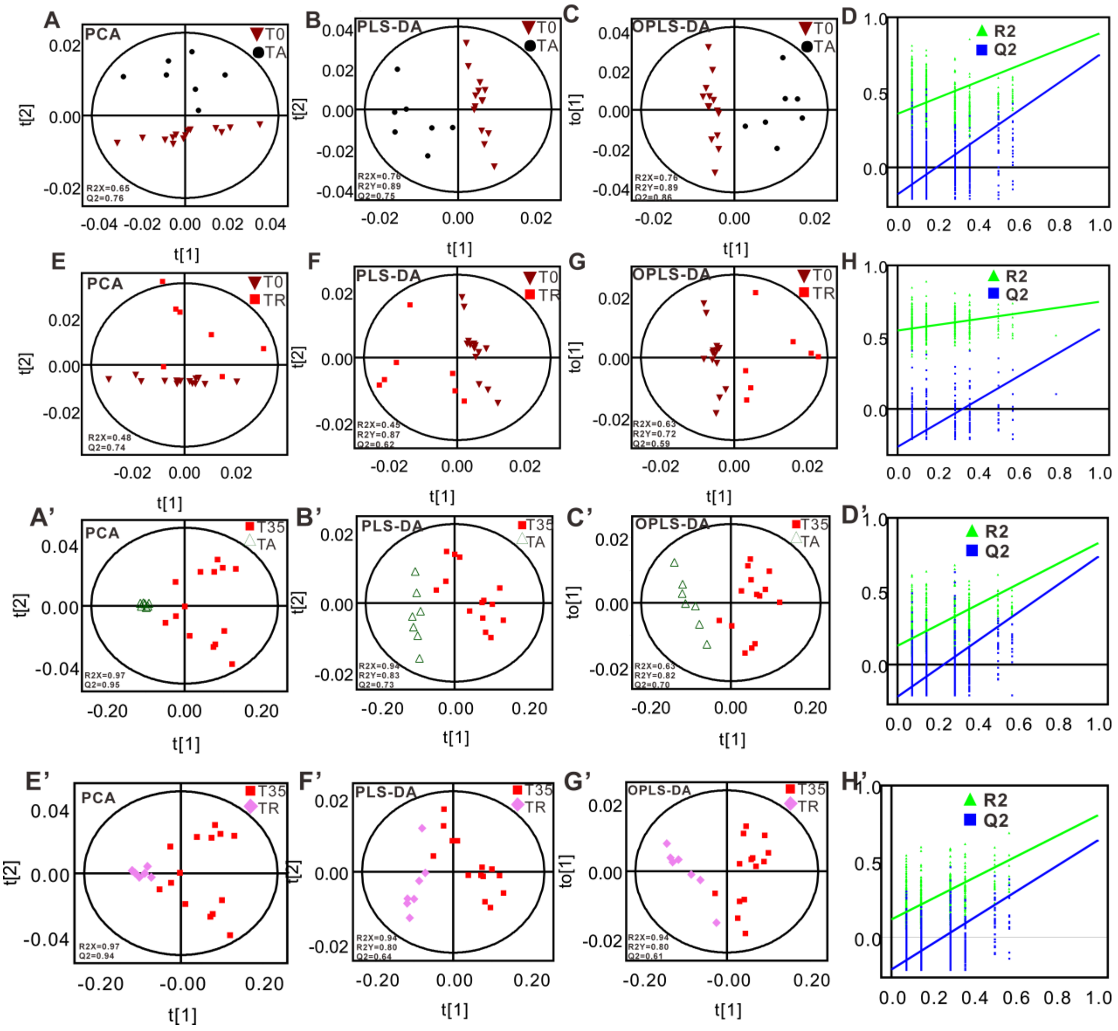

Figure S5. OPLS-DA coefficient plots of ^1H NMR data from TA vs. T0 groups (A), TR vs. T0 groups (B). The abbreviations of metabolites are denoted in Table S1.

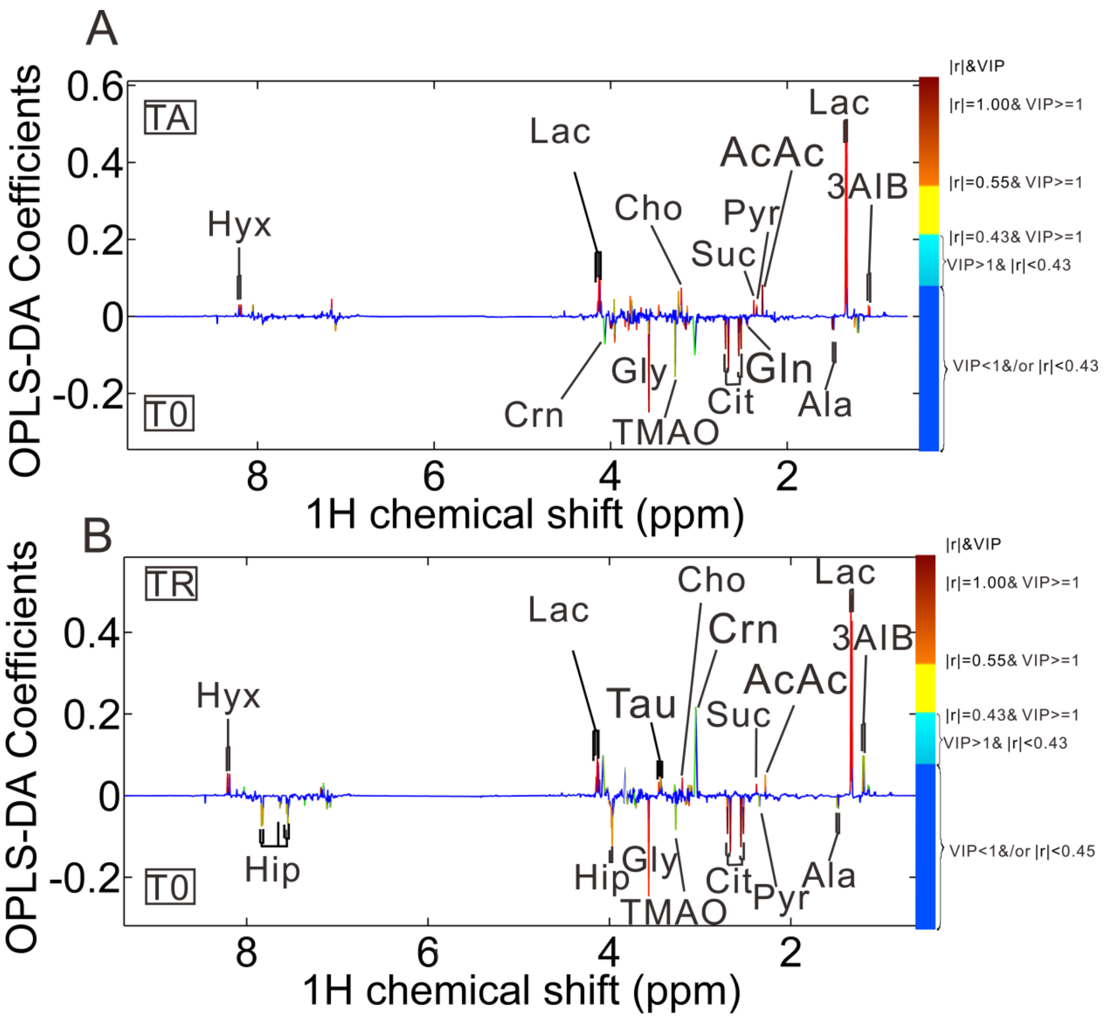

Table S1. Resonance assignments of metabolites in ^{1}H NMR spectra.

Metabolite (abbreviation)	Groups	δ ^{1}H(ppm) in PBS buffer (pH=7.2) #
2-hydroxyisovalerate(2HIV)	α-CH, β-CH, γ-CH_3, γ-CH_3	3.80(d), 2.00(m), 0.96(d), 0.82(d)
2-hydroxybutyrate(2HB)	α-CH, half β-CH_2, half β-CH_2, δ-CH_3	3.98(m), 1.74(m), 1.66(m), 0.90(t)
isocaproate(IC)	α-CH_2, β-CH_2, γ-CH, 2×(δ-CH_2)	0.86(d), 2.18(m), 1.45(m), 1.50(m)
leucine(Leu)	α-CH, β-CH_2, δ-CH_3, δ-CH_3	3.72(m), 1.73(m), 1.70(m), 1.69(m), 0.98(d), 0.97(d)
isoleucine(Ile)	α-CH, β-CH, γ-CH_3, half γ-CH_2, half γ-CH_2, δ-CH_3	3.67(d), 2.05(m), 1.02(d), 1.45 (m), 1.23(m), 0.96(t)
valine(Val)	α-CH, β-CH, γ-CH_3, γ-CH_3	3.61(d), 2.27(m), 1.06(d), 1.01(d)
2-aminobutyrate(2AB)	α-CH, β-CH_2, γ-CH_3	3.70(t), 1.90(m), 0.98(t)
isobutyrate(IB)	α-CH, 2×β-CH_3	2.35(m), 1.08(d)
3-aminoisobutyrate(3AIB)	α-CH, β-CH_3, half β-CH_2, half β-CH_2	2.60(m), 1.19(d), 3.02(q), 3.09(q)
3-hydroxyisovalerate(3HIV)	α-CH_2, 2×γ-CH_3	1.27(s), 2.35(s)
lactate(Lac)	α-CH, β-CH_3	4.13 (q), 1.38(d)
2-hydroxyisobutyrate(2HIB)	2×β-CH_3	1.33(s)
analine(Ala)	α-CH, β-CH_3	3.78(q), 1.49(d)
N-acetyl-glucoprotein(NAG)	α-CH_2	2.00(s)
glutamine (Gln)	α-CH, β-CH_2, γ-CH_2	3.78(t), 2.44(m),

		2.14(m)
pyruvate(Pyr)	CH_3	2.36(s)
succinate(Suc)	2×CH_2	2.41(s)
citrate(Cit)	half α-CH_2, half α-CH_2	2.55(d), 2.68(d)
2-dimethylamine(DMA)	CH_3	2.72(s)
methylguanidine(Mgu)	CH_3	2.82(s)
trimethylamine(TMA)	$N(CH_3)_3$	2.86(s)
dimethylglycine(DMG)	α-CH_2, $N(CH_3)_2$	3.71(s), 2.93(s)
creatinine(Crn)	α-CH_2, N-CH_3	4.03(s), 3.05(s)
Trimethylamine N-oxide(TMAO)	CH_3	3.27(s)
taurine(Tau)	$^1CH_2$, $^2CH_2$	3.43(t), 3.27(t)
glycine(Gly)	α-CH_2	3.57(s)
fumarate(FMA)	2×CH	6.53(s)
tyrosine(Tyr)	phenyl moiety: α-CH, β-CH, half β-CH_2, half β-CH_2	7.19(d), 6.92(d), 3.05(dd), 3.19(dd)
phenylacetylglycine(PAG)	α-CH, β-CH, γ-CH, CH_2(CONH), CH_2(COOH)	7.34(d), 7.41(t), 7.34(t), 3.66(s), 3.74(d)
hippurate(Hip)	α-CH, β-CH, γ-CH, CH_2	7.82(d), 7.54(t), 7.62(t), 3.96(d).
hypoxanthine(Hyx)	γ-CH, β-CH,	8.17(s), 8.20(s)
formate(For)	CH	8.46(s)
trigonelline(TRG)	α'-CH, α-CH, γ-CH, β-CH, CH_3	9.11(s), 8.82(d), 8.81(d),

		8.08(t) ,4.42(s)
N-methylnicotinamide (NMN)	α-CH, α-CH, γ-CH, β-CH, CH_3N.	9.27(s), 8.95(d), 8.88(d), 8.18(m), 4.47(s)

[#] s, singlet; d, doublet; t, triplet; q, quartet; m, many peaks.

Table S2. Quantitative integrals of several discriminitive metabolites found in the urine samples of T0, T35, TA, and TR groups.

Metabolites	Integrals in T0 group (mean±std)×10^{-3}	Integrals in T35 group (mean±std)×10^{-3}	Integrals in TA Group (mean±std)×10^{-3}	Integrals in TR group (mean±std)×10^{-3}
2HB	0.31±0.04	1.06±0.38#	0.37±0.04	0.46±0.13
Lac	1.23±0.87	96.88±34.38*	6.85±4.32	12.79±9.49
Ala	1.18±0.61	1.85±0.51#	0.87±0.10	1.01±0.42
Gln	1.01±0.27	0.66±0.33#	0.55±0.34	0.52±0.35
Pyr	0.97±0.68	0.77±0.35#	1.28±0.81	0.74±0.32
Suc	0.55±0.08	8.46±4.85#	1.24±0.32	1.22±0.50
Cit	3.00±1.41	1.08±0.41#	1.99±1.22	1.31±0.40
DMA	2.30±0.36	2.14±0.21#	2.25±0.21	2.64±0.41
DMG	1.32±0.32	0.93±0.20#	1.18±0.38	1.06±0.30
Crn	111.11±13.46	113.30±13.71#	110.02±9.32	120.62±9.41
TMAO	2.71±1.51	1.85±0.73#	2.04±0.70	2.50±0.68
Tau	0.90±0.39	0.64±0.28#	0.93±0.44	1.49±0.90
Gly	6.72±3.86	2.68±1.00#	3.15±1.19	2.74±1.21
Hip	1.66±1.53	1.30±1.39#	1.35±0.95	0.49±0.31
Hyx	0.06±0.04	1.21±0.52#	0.43±0.37	0.81±0.49
For	0.57±0.37	0.17±0.10#	0.29±0.14	0.24±0.08

# The relative integrals of metabolites were determined from 1D ^{1}H NMR spectra in which the signals of lactate were excluded.

* The relative integral of lactate was determined from 1D ^{1}H NMR spectra in which the signals of lactate were included.
